# A multiparametric analysis including single-cell and subcellular feature assessment reveals differential behavior of spheroid cultures on distinct ultra-low attachment plate types

**DOI:** 10.3389/fbioe.2024.1422235

**Published:** 2024-08-02

**Authors:** Mario Vitacolonna, Roman Bruch, Ane Agaçi, Elina Nürnberg, Tiziana Cesetti, Florian Keller, Francesco Padovani, Simeon Sauer, Kurt M. Schmoller, Markus Reischl, Mathias Hafner, Rüdiger Rudolf

**Affiliations:** ^1^ CeMOS, Mannheim University of Applied Sciences, Mannheim, Germany; ^2^ Institute of Molecular and Cell Biology, Mannheim University of Applied Sciences, Mannheim, Germany; ^3^ Institute for Automation and Applied Informatics, Karlsruhe Institute of Technology, Karlsruhe, Germany; ^4^ Faculty of Biotechnology, Mannheim University of Applied Sciences, Mannheim, Germany; ^5^ Institute of Functional Epigenetics (IFE), Molecular Targets and Therapeutics Center (MTTC), Helmholtz Center München, München-Neuherberg, Germany; ^6^ Institute of Medical Technology, Medical Faculty Mannheim of Heidelberg University and Mannheim University of Applied Sciences, Mannheim, Germany

**Keywords:** CCD-1137Sk, cytokeratin-14, CK14, HaCaT, HT-29, Involucrin, Ki-67, MDA-MB-231

## Abstract

Spheroids have become principal three-dimensional models to study cancer, developmental processes, and drug efficacy. Single-cell analysis techniques have emerged as ideal tools to gauge the complexity of cellular responses in these models. However, the single-cell quantitative assessment based on 3D-microscopic data of the subcellular distribution of fluorescence markers, such as the nuclear/cytoplasm ratio of transcription factors, has largely remained elusive. For spheroid generation, ultra-low attachment plates are noteworthy due to their simplicity, compatibility with automation, and experimental and commercial accessibility. However, it is unknown whether and to what degree the plate type impacts spheroid formation and biology. This study developed a novel AI-based pipeline for the analysis of 3D-confocal data of optically cleared large spheroids at the wholemount, single-cell, and sub-cellular levels. To identify relevant samples for the pipeline, automated brightfield microscopy was employed to systematically compare the size and eccentricity of spheroids formed in six different plate types using four distinct human cell lines. This showed that all plate types exhibited similar spheroid-forming capabilities and the gross patterns of growth or shrinkage during 4 days after seeding were comparable. Yet, size and eccentricity varied systematically among specific cell lines and plate types. Based on this prescreen, spheroids of HaCaT keratinocytes and HT-29 cancer cells were further assessed. In HaCaT spheroids, the in-depth analysis revealed a correlation between spheroid size, cell proliferation, and the nuclear/cytoplasm ratio of the transcriptional coactivator, YAP1, as well as an inverse correlation with respect to cell differentiation. These findings, yielded with a spheroid model and at a single-cell level, corroborate earlier concepts of the role of YAP1 in cell proliferation and differentiation of keratinocytes in human skin. Further, the results show that the plate type may influence the outcome of experimental campaigns and that it is advisable to scan different plate types for the optimal configuration during a specific investigation.

## 1 Introduction

Three-dimensional cell cultures, such as spheroids and organoids, provide intermediate complexity and relevance as biological model systems for fundamental and applied research inquiries (Alemany-Ribes and Semino, 2014). Compared to classical adherent monolayer cell cultures, 3D models can better reflect naturally occurring gradients of drugs, waste, nutrients, and gases than two-dimensional cultures and they usually better allow assessing the effects of extracellular matrix, cellular interactions, and drugs (Fontoura et al., 2020; Bär et al., 2022). To harness the full analytical power of 3D-cell cultures, current studies often use a combination of live-cell morphometry and subsequent end-point measurements. While the former is good for higher throughput prescreens to reveal conditions or time points of interest for deeper investigation (Phillip et al., 2021; Zhang et al., 2023), the latter may serve to get mechanistic insights, for example regarding a drug’s mechanism of action or concerning processes underlying cellular differentiation (Single et al., 2015; Nunes et al., 2024; Rahman et al., 2024). At present, single-cell technologies, such as droplet microfluidics combined with single-cell transcriptomics or fluorescence microscopy of optically cleared samples combined with single-cell 3D-image analysis are at the analytical spearhead to either maximize the information content towards molecular detail or towards the correlation between cell location and cell function/identity (Delage et al., 2023; Li et al., 2023; Jiang et al., 2024). Regarding 3D-image segmentation in 3D-cell cultures, segmentation and quantification of nuclei has so far received the most attention and different studies have provided tools for this purpose and quantitative data under several experimental conditions (Barbier de Reuille et al., 2015; Belevich and Jokitalo, 2021; Padovani et al., 2022). One study also looked at substructures within nuclei (Bokota et al., 2021). Due to its complexity, the 3D analysis of cell shape in 3D-cell cultures has been solved in very few studies to investigate the deformation of cell nuclei in osteocytic spheroids (Inagaki et al., 2023) or to analyze the morphology of over 95,000 melanoma cells, offering a new way to understand cellular shape in three dimensions (Vries et al., 2023). However, the automated combined analysis of more than one subcellular structure, e.g., of cytoplasm and nucleus, at the single-cell level of a 3D-cell culture has not been addressed to our knowledge. In particular, such an examination could shed light on the relative cytoplasmic-nuclear distribution of transcription factors and transcriptional coregulators that shuttle between both compartments as a proxy of their activity. Together with its paralog, TAZ, YAP is a transcriptional coregulator downstream of the Hippo pathway, which controls cell proliferation and survival, metabolism and motility, as well as cell fate and differentiation as a function of mechanical signals (Ma et al., 2019). Factors including cell-cell contacts, ECM stiffness, cell shape and stretching (Ma et al., 2019) control Hippo activity, whereby the downstream kinases, LATS1 and LATS2, mediate YAP phosphorylation, cytoplasmic sequestration, and inactivation of YAP-dependent gene expression. Hippo signaling is critically altered in several imbalance and disease states, such as in wound healing and cancer (Dey et al., 2020) and it has been intensely addressed as a drug target (Dey et al., 2020). Yet, due to its pleiotropic regulation, YAP activity and function were found to be significantly different between adherent and 3D-cell cultures (Lee et al., 2019; Jahin et al., 2023; Oliva-Vilarnau et al., 2023), arguing for further research in that direction. Since YAP nuclear translocation is a major proxy for YAP activity (Ma et al., 2019; Zou et al., 2020), the determination of the YAP nuclear-to-cytoplasm ratio (N/C ratio) is key to single-cell assays in this field. However, automated analysis of this characteristic in 3D samples has been hampered by technical issues and it has been achieved only for very small spheroids (Lee et al., 2019) or by manually checking a few cells in larger spheroids (Jahin et al., 2023).

Among the currently available 3D-culture models, spheroids have likely been most frequently used due to their relative ease of production and their reproducibility in terms of key features, such as size and response to drugs (Nath and Devi, 2016). Spheroids are mostly made of immortal cell lines and are composed of a single or a few different cell types, depending on the addressed question. Spheroid production may employ scaffolding substrates, such as collagen or Matrigel, or may be achieved in a scaffold-free manner (Nath and Devi, 2016; Rustamov et al., 2017; 2019; Keller et al., 2019; 2021). Typically, regardless of the use of scaffolds or not, the creation of spheroids is based on avoiding the attachment of cells to any other surface than neighboring cells. Therefore, techniques such as hanging drop, bioreactor culture, matrix encapsulation, magnetic levitation, or ultra-low attachment (ULA) plates have been devised (Nath and Devi, 2016). The high technical fidelity, that can normally be obtained with spheroid cell cultures allows for determining even subtle effects and/or testing several experimental conditions with acceptable efforts in workload, time, and materials. To achieve maximal technical robustness, all components in the testing pipeline need to perform optimally and reliably. Previous studies addressed the effects of different spheroid-generation types (Bresciani et al., 2019; Wu et al., 2021), substrates (Serrati et al., 2020), surfaces (Azizipour et al., 2022), use of microfluidics (Azizipour et al., 2022), and media volume (Das et al., 2016) on the robustness of spheroid formation. Due to their ease of use for multiple drug testing purposes, ULA plates have been of increasing relevance among the spheroid-generation modes. Several commercial ULA-plate products with similar base technology, using mostly 96-well and 384-well standard plate formats, are currently available. Although differences in their capability on spheroid formation and growth can be anticipated and although this knowledge could be of interest in terms of experimental planning and regulatory aspects, a systematic analysis of different plate layouts on the formation and development of spheroids has not been published, to our knowledge.

The present study aimed at extending 3D-single cell analysis towards the determination of the N/C-ratio of the transcription factor, YAP. Therefore, a pipeline from spheroid generation over optical tissue clearing, 3D-fluorescence staining, and 3D-confocal microscopy, to automated 3D-segmentation and co-registration of cytoplasm and nuclei was developed. To identify relevant experimental conditions in the context of YAP regulation, spheroids from 4 cell lines with different growth and differentiation characteristics were grown in six distinct 96-well ULA plate types and first prescreened morphologically in live state regarding spheroid formation, roundness, compactness, and growth. In detail, the non-neoplastic foreskin fibroblast cell line, CCD-1137Sk, and the keratinocyte cell line, HaCaT, were used as models with low proliferative activity in 3D (Klicks et al., 2019). HaCaT cells were also selected, because of their capacity to display cellular differentiation (Klicks et al., 2019). Further, colon cancer cells, HT-29, and breast cancer cells, MDA-MB-231, were used as representatives for highly proliferative cell types, that are either easily forming spheroids in the absence of any scaffold (HT-29) (Kwok et al., 1988; Keller et al., 2020) or that are dependent on the addition of ECM-components for efficient spheroid assembly (MDA-MB-231) (Froehlich et al., 2016; Rustamov et al., 2019; Keller et al., 2021). Briefly, although all tested plate types consistently led to the formation of spheroids, showing the maturity and reliability of this technological platform, the live cell results displayed interesting differences in simple morphological terms for HaCaT and HT-29 spheroids when comparing the distinct plate types. Consequently, these were subjected to the high-content single-cell fluorescence microscopy pipeline. In HaCaT keratinocyte spheroids, which were known to stratify into more and less differentiated cells, the combined 3D-single cell analysis of cell number, Ki-67 positive cells, YAP expression and distribution, and differentiation markers revealed a correlation between cell proliferation, differentiation, and YAP N/C ratio in a spatially defined manner.

## 2 Materials and methods

### 2.1 Cell culture and spheroid generation

To investigate the influence of the cell culture plates on the generation and growth of spheroids, 96-well ULA plates from six different manufacturers were tested (in the following referred to as A-F): A: BIOFLOAT™ 96-well plates (faCellitate, #F202003), B: BRANDplates^®^ 96-well microtitration plate (BrandTech Scientific, #781900), C: Cellstar^®^ 96-well Microplate (Greiner, #650970), D: CellCarrier Spheroid ULA 96-well Microplates (PerkinElmer, #6055330), E: Corning^®^ Costar^®^ 96-well Clear Round Bottom Ultra-Low Attachment Microplates (Corning, #7007), F: 96-well plate Sphera™ Low-Attachment Surface (ThermoScientific, #174927). The following 4 cell lines were used to generate spheroids: CCD-1137Sk human foreskin fibroblast cells (ATCC, CRL-2703) were cultured in Iscove’s modified Dulbecco’s medium (IMDM, Capricorn, IMDM-A) supplemented with 10% fetal bovine serum (FBS, Capricorn, FBS-16B) and 1% penicillin/streptomycin (Pen/Strep, Sigma-Aldrich, P4333). For spheroid monoculture generation, cells were detached using Trypsin/EDTA (Sigma-Aldrich, T4174) and seeded onto 96-well ULA plates at a concentration of 2 × 10^3^ cells per well. The human keratinocyte cell line HaCaT (kindly provided by BRAIN AG, Zwingenberg) was cultured in Dulbecco’s Modified Eagle Medium (DMEM) High Glucose with L-Glutamine and Sodium Pyruvate (Capricorn, DMEM-HPA) supplemented with 1% Pen/Strep and 10% FBS. For spheroid generation, cells were detached using Trypsin/EDTA and seeded onto the ULA plates at a concentration of 5 × 10^3^ cells per well. HT-29 colon cancer cells (ATCC, HTB-38) were cultured in McCoy’s 5A medium (Capricorn, MCC-A) supplemented with 10% FBS and 1% Pen/Strep. For spheroid generation, cells were detached using Trypsin/EDTA and seeded onto the ULA plates at a concentration of 5 × 10^2^ cells per well. MDA-MB-231 cells (ATCC, CRM-HTB-26) were cultured in RPMI 1640 medium with L-glutamine (Capricorn, RPMI-A) supplemented with 10% FBS and 1% Pen/Strep. For spheroid generation, cells were detached using Trypsin/EDTA. Cell suspension was supplemented with 5 mM type 1 collagen (Roche Diagnostics, 11179179001) to allow spheroid formation and seeded onto the ULA plates at a concentration of 5 × 10^3^ cells per well. Each cell line was seeded in the appropriate plate type as replicates (n = 24 per cell line), and all experiments were conducted as three biological replicates. All cells were maintained in a humidified incubator at 37°C with 5% CO_2_ fumigation.

### 2.2 Brightfield imaging and measurement of spheroid diameter, eccentricity, and area covered by dissociated cells

Plates were imaged every 24 h for 4 days using the Cytation™ 5 cell imaging multi-mode plate reader with BioSpa 8 automated incubator, using a ×10 phase contrast objective and Gen5 software (all BioTek Instruments). Spheroid diameter and eccentricity were automatically quantified using MATLAB with the SpheroidSizer software (Chen et al., 2014). To measure the area covered by dissociated cells around the HaCaT spheroids, we used a two-step approach due to the inhomogeneous intensity distribution of the objects to be measured. First, a pixel-based classification was performed using the machine-learning-based bio-image analysis tool Ilastik (V1.4.0) (Berg et al., 2019). The pixel classifier aims to learn to distinguish whether each pixel belongs to a specific object type or background, using not only the intensity information of that pixel but also the intensity information of local pixel neighbors (Sommer and Gerlich, 2013). Two classes (background, cells) were defined and manually labeled separately using the paintbrush tool, which was used to train Ilastik’s machine-learning algorithm to identify the objects of interest. The training was iterative, adding new pixel classifications until the probability maps were stable and adequately distinguished the object types. The training was repeated for each plate type, as the background of each plate type was different. The pixel classification workflow described here performed a semantic segmentation that divides the image into two semantic classes (foreground and background), but not into individual objects. For each pixel in the image, Ilastik estimates the probability that the pixel belongs to each of the semantic classes. The resulting probability maps were then exported as .tif files and loaded into the open-source software CellProfiler (V4.2.6) (Carpenter et al., 2006) in combination with the corresponding raw brightfield images to perform segmentation of the dissociated cells. The probability maps were converted to a single channel using the “ColortoGray” module to obtain only the cell channel. The images were then smoothed with a 5-pixel wide Gaussian filter and segmented using the IdentifyPrimaryObjects module using Otsu’s method with two-class thresholding and an object diameter between 5 and 200 pixels to exclude the core spheroid. The segmented objects were then quantified using the “MeasureObjectSizeShape” and “MeasureImageAreaOccupied” modules. The “CalculateMath” and “DisplayDataOnImage” modules were used to calculate the percentage of occupied area and to create overlays.

### 2.3 Wholemount immunostaining and optical clearing

Spheroids (n = 10 per group) were transferred to Eppendorf tubes, washed once with phosphate-buffered saline (PBS, Sigma Aldrich, P2272), and fixed with 4% paraformaldehyde (PFA, Carl Roth, 0335.3) for 1 h at 37°C, followed by two washes with PBS containing 1% FBS for 5 min each. To remove traces of fixative, spheroids were quenched with 0.5 M glycine (Carl Roth, 3187.3) in PBS for 1 h at 37°C with gentle shaking. Spheroids were then incubated for 30 min in a penetration buffer containing 0.2% Triton X-100 (3051.2), 0.3 M glycine, and 20% DMSO (AE02.1) (all Carl Roth) in PBS to enhance the penetration of antibodies and nuclear stains. Spheroids were then incubated in a blocking buffer [0.2% Triton X-100, 1% BSA (Carl Roth, 8076.3), 10% DMSO in PBS] for 2 h at 37°C with gentle shaking. After blocking, samples were incubated with primary antibodies overnight (ON) at 37°C with gentle shaking. Primary antibodies were diluted in antibody buffer (0.2% Tween 20 (11332465001), 10 μg/mL heparin (9041-08-1), both Sigma-Aldrich, 1% BSA, 5% DMSO in PBS) at the following concentrations: mouse anti-Ki-67 1:300 (Abcam, [B56], ab279653), rabbit anti-YAP1 1:150 (Invitrogen, PA1-46189). Samples were then washed 5 x for 10 min each in wash buffer (0.2% Tween-20, 10 μg/mL heparin, 1% BSA) and stained with secondary antibodies goat anti-mouse IgG (H + L) Alexa Fluor^®^488 1:500 (Invitrogen, A-11001), donkey anti-rabbit IgG (H + L) Alexa Fluor^®^555 1:800 (Invitrogen, A32794), SiR-actin 1:1,000 (Spirochrome, SC001) and DAPI 1:1,000 (Sigma-Aldrich, 10236276001) ON at 37°C in antibody buffer with gentle shaking. Samples were then washed 5 x for 10 min in washing buffer with gentle shaking and cleared with FUnGI clearing solution [50% glycerol (vol/vol) (3783.3), 2.5 M fructose (4981.4), 2.5 M urea (2317.1), 10.6 mM Tris Base (4855.2), 1 mM EDTA (1P17.1]; all Carl Roth) ON as previously described (van Ineveld et al., 2020). Cleared samples were transferred to 18 well µ-slides (Ibidi, 81816) in the same solution and kept in the microscope room for several hours to allow for temperature adjustment.

### 2.4 Spheroid cryosectioning and staining

HaCaT spheroids were collected in an Eppendorf tube for cryosectioning. After being washed twice with PBS, they were fixed with 4% paraformaldehyde in PBS for 30 min at room temperature. Following this, the spheroids were incubated in 15% sucrose (Carl Roth, 4621.1) in PBS overnight at 4°C, then in 25% sucrose in PBS again overnight at 4°C. Subsequently, they were embedded in Tissue-Tek Cryomolds using OCT (Leica Biosystems). 15-μm thick sections were prepared using a CM-1950 cryostat (Leica Biosystems). Cryosections were permeabilized with 0.1% Triton X-100 (Carl Roth) in PBS, then blocked with 2% BSA in PBS before being stained with rabbit anti-Cytokeratin 10 1:1,000 (Abcam; ab76318), rabbit anti-Cytokeratin 14 1:1,000 (Thermo-Fisher, PA5-16722), and mouse anti-Involucrin 1:1,000 (Abcam; ab20202) ON at 4°C. Samples were washed 3 x with PBS containing 1% FBS, followed by secondary antibody and nuclei staining using donkey anti-rabbit IgG (H + L) Alexa Fluor^®^488 1:800 (Thermo-Fisher, A32790), anti-mouse IgG (H + L) Alexa Fluor^®^555 1:800 (Thermo-Fisher, A-21424), and DAPI 1:1,000 for 2 h at RT. Finally, sections were washed 3 x with PBS/1% FBS, mounted with Mowiol (Carl Roth, 0713.2) and imaged using a confocal microscope (SP8, Leica).

### 2.5 Image acquisition using confocal microscopy

All 3D cultures and cryosections were imaged using an inverted Leica TCS SP8 confocal microscope (Leica Microsystems CMS, Mannheim, Germany) equipped with an HC PL APO 20×/0.75 IMM CORR objective, 488 nm, 561 nm and 633 nm lasers and Leica Application Suite X software. 3D-wholemount image stacks were acquired with comparable settings, using Immersion Type F (Leica Microsystems, RI 1.52) as immersion fluid, with a resolution of 1,024 × 1,024 pixels (473 × 473 nm per pixel), a z-step size of 1 μm, a laser intensity of 1%–1.5% and a gain setting of 600 to avoid overexposure of pixels. All image stacks were acquired with z-compensation to compensate for depth-dependent signal loss. Cryosections were imaged with a resolution of 1,024 × 1,024 pixels (473 × 473 nm per pixel), a z-step size of 1 μm, a laser intensity of 0.4%–1.2% and a gain setting of 600 to avoid overexposure of pixels.

### 2.6 3D-segmentation and image analysis

Raw confocal data were converted to multi-channel .tif files using Fiji (Schindelin et al., 2012). 3D segmentation of nuclei and fluorescence signals of Ki-67 and plasma membrane (SiR-actin) staining was performed using Cellpose (V2.2), a deep learning-based instance segmentation tool (Stringer et al., 2021). For each cell type and fluorescence marker, the convolutional neural network was trained on hand-annotated ground truth datasets prepared from nuclei, Ki-67, and plasma membrane datasets to improve segmentation accuracy. To prepare the annotated training data, spheroids of each cell type were first pre-segmented using the pre-trained nuclei and cyto2 models in Cellpose, including the two fluorescent markers for DAPI and Ki-67, as an initial step. From these, three patches with sizes of 32 × 128 × 128 pixels (z, y, x) were extracted for each and manually corrected using the Segmentor software (Borland et al., 2021). Supervised training from scratch was performed as described in (Pachitariu and Stringer, 2022) using the command line interface for Cellpose. We varied the number of training epochs from 50 to 1,000 and tested the segmentation performance with the segmentation (SEG) and detection (DET) measures used in the cell tracking challenge (Ulman et al., 2017). Custom-trained models with the highest DET score were selected for the subsequent segmentation of the 3D datasets. To perform the segmentation and subsequent quantitative analysis in batch mode, we used Cell-ACDC (V1.4), an open-source graphical user interface (GUI)-based framework for cell segmentation, visualization, and data analysis that embeds various neural networks such as Cellpose (Padovani et al., 2022). For segmentation, the appropriate custom-trained model was loaded into Cellpose with the following parameters: “flow_threshold = 0.4,” “Cellprob_threshold = −2.0,” and “stich_threshold = 0.7” for nuclei and Ki-67 channels, and “stich_threshold = 0.3” for the f-actin channel. The cell diameters were automatically calculated by the algorithm. The output label masks were then used for downstream analysis such as quantification of object count and volumes using the “regionprops” function from the “scikit-image” Python package built into Cell-ACDC. Segmented nuclei with a volume of less than 300 μm^3^ and greater than 3,000 μm^3^ were considered debris or segmentation errors and excluded from further analysis. Quantitative analysis of spheroid volume and density was performed using dedicated Python scripts. The nuclei segmentation results were used as a starting point. This was followed by 40 iterations of binary dilation followed by 40 iterations of binary erosion to close the holes between the nuclei without increasing the overall size of the spheroid segmentation. A structuring element with a connectivity of 1 was used. The remaining holes within the spheroid segmentation were filled. If several unconnected structures remained, only the largest was used. Spheroid density was calculated by dividing the number of nuclei inside the spheroid by the volume of the segmented spheroid. The void region inside the spheroid was defined as the volume of the segmented spheroid excluding the nuclei segmentation, i.e., the region outside the nuclei and within the spheroid segmentation, in other terms, they should largely represent cytoplasm plus non-nuclear organelles plus extracellular space. To calculate the relative number of proliferative cells, the amount of Ki-67^+^ cells was divided by the total number of nuclei counted.

### 2.7 Calculation of the YAP N/C ratio

To calculate the YAP1 N/C ratio at the single-cell level, we used the “Track sub-cellular objects” tool integrated into Cell-ACDC. This tool allows for the flexible selection of the minimum percentage overlap (Intersection over Union, IoU) between nuclei and membrane segmentation masks in 3D datasets to associate the objects. For our analysis, we selected a minimum IoU of 50% (IoU ≥ 0.5). As a result of the subsequent subtraction of the segmentation masks for membranes and nuclei, a third segmentation file was generated with the cytoplasm segmentation masks (with the same IDs of the corresponding membrane and nuclei masks). Subsequently, the newly generated nuclear and cytoplasmic segmentation masks with matching ID numbers were used to measure YAP1 intensities separately in the nuclei and cytoplasm segments in Cell-ACDC. The amount of YAP1 was measured as the mean (the sum of all pixel intensities divided by the volume), incorporating an automatic background correction (defined as all pixels outside the detected objects). Based on these mean values, the N/C ratio was calculated for all groups. Similarly, the segmentation masks for Ki-67 were aligned with the membrane label identifiers, enabling subsequent analyses of the N/C ratio in both Ki-67+ and Ki-67- cells.

### 2.8 Spatial analysis of cell populations

A custom Python-based image analysis pipeline was developed to quantify cellular properties from membrane, nuclear and cytoplasmic label masks, as well as raw microscopy images. The pipeline integrated several key libraries, including NumPy for array manipulations, Pandas for data handling, scikit-image for image processing, SciPy for scientific computing, and Matplotlib and Seaborn for data visualization. For each segmented cellular component, geometric features (e.g., centroid coordinates, volume) and fluorescence intensity features, from which the YAP1 N/C ratio was calculated, were extracted. Additionally, each label was categorized as either Ki-67+ or Ki-67-, based on the co-occurrence of a Ki-67 label sharing the same ID. To determine the spatial distribution of cells inside the spheroid, a convex hull was constructed around the centroids of all identified cellular components within the image, effectively outlining the outer boundary of the cellular distribution. For each cell, a line was constructed, passing through the centroid of the cell and the center of the convex hull. The intersection of this line with the spheroid hull was then utilized to calculate the distance between the spheroid hull and the cell’s centroid. Scatter matrix plots were generated to visually assess the multidimensional relationships between cellular properties and marker expressions.

### 2.9 Cryosection image analysis

Images from cryosections were converted to .tif files with Fiji. The three most in-focus z-planes were summed and used for further analysis. The spheroid area was obtained by selecting the whole spheroid in the Involucrin channel with the wand tracing tool. After median filtering with a radius of 2, a segmentation was obtained for Involucrin and CK14 signals, based on thresholding. In these segments, the mean intensity and the area were measured and used to calculate the Integrated Density (mean intensity x normalized area; the area was normalized to the spheroid area). The ratio between the Integrated Densities of the Involucrin and of the CK14 signals was calculated for each cryosection.

### 2.10 Statistical analysis

The statistical analyses in this study were conducted using GraphPad Prism 9, ensuring all data underwent tests for normal distribution. To compare the results between brightfield and 3D immunostainings, we employed an ordinary one-way ANOVA, incorporating Šidák’s correction for multiple comparisons. We established a significance threshold (α) at 0.05, corresponding to a 95% confidence interval. For the analysis of 2D immunostainings, the difference in means was assessed using Student’s t-test. In instances where pairs of groups deviated from normal distribution, we used the non-parametric Mann-Whitney U test to determine statistical significance.

## 3 Results

### 3.1 Fibroblast spheroids form in all plate types but show systemic differences in size

**FIGURE 1:**
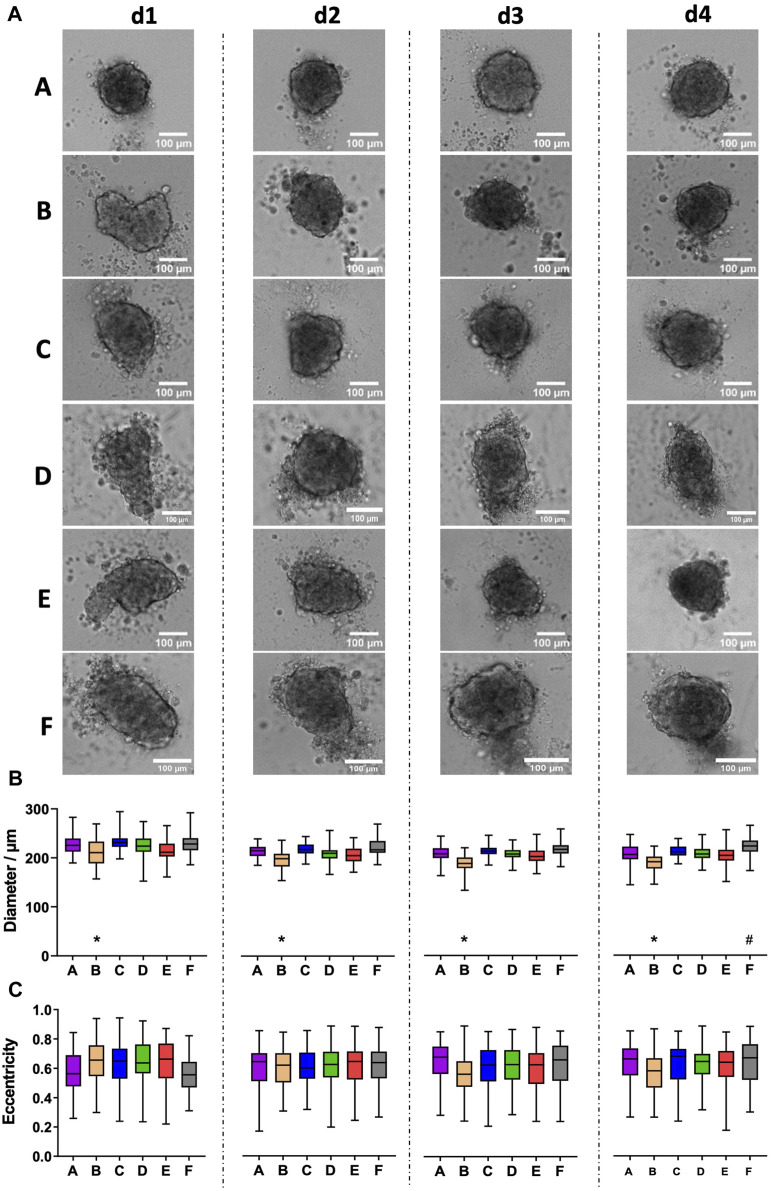
CCD-1137Sk fibroblast spheroids vary in size between different plate types. Freshly trypsinized CCD-1137Sk cells were seeded into 96-well ULA plates, types A–F, at a density of 2,000 cells per well and then cultured for up to 4 days. Spheroid morphology was visualized daily using automated brightfield microscopy. **(A)** Representative micrographs showing individual spheroids from day 1–4 (d1-d4) in plate types A–F. **(B, C)** Box-Whisker plots depicting spheroid diameters **(B)** or eccentricity **(C)** as a function of plate type A–F. Data are from three experiments with ≥ 24 spheroids per experiment (box: median, lower and upper quartiles; whiskers: Min to Max). Complete significance analysis, see Supplementary Figure S1. * and #, values significantly different compared to all other plate types for samples from the same day.

CCD-1137Sk human foreskin fibroblast cells were taken from freshly trypsinized adherent cultures and simultaneously seeded into 96-well plates of types A-F with 2,000 cells per well. Brightfield images were taken daily and for 4 days after seeding using an automated microscope. Often irregularly shaped spheroids formed in all tested plate types, and comparable amounts of loose cells were detected in all plates (Figure 1A). On average, quantitative image analysis showed a slight decrease in spheroid diameters from day 1 to day 2 after seeding, but then, diameters remained rather stable (Figure 1B). As anticipated from the irregular shape of most spheroids, eccentricity was at a relatively high value of around 0.6 throughout the entire observation time (Figure 1C). Eccentricity and size variations among spheroids were similar for all plate types. Conversely, there were consistent differences in spheroid diameters: First, spheroids of plate type B were smaller than those of all other types during the full experimental time window of 4 days (Figure 1B, asterisks). Second, on day 4, spheroids of plate type F were larger than those of all other types (Figure 1B, hashtag).

### 3.2 Formation of MDA-MB-231 spheroids is variable between plate types

**FIGURE 2:**
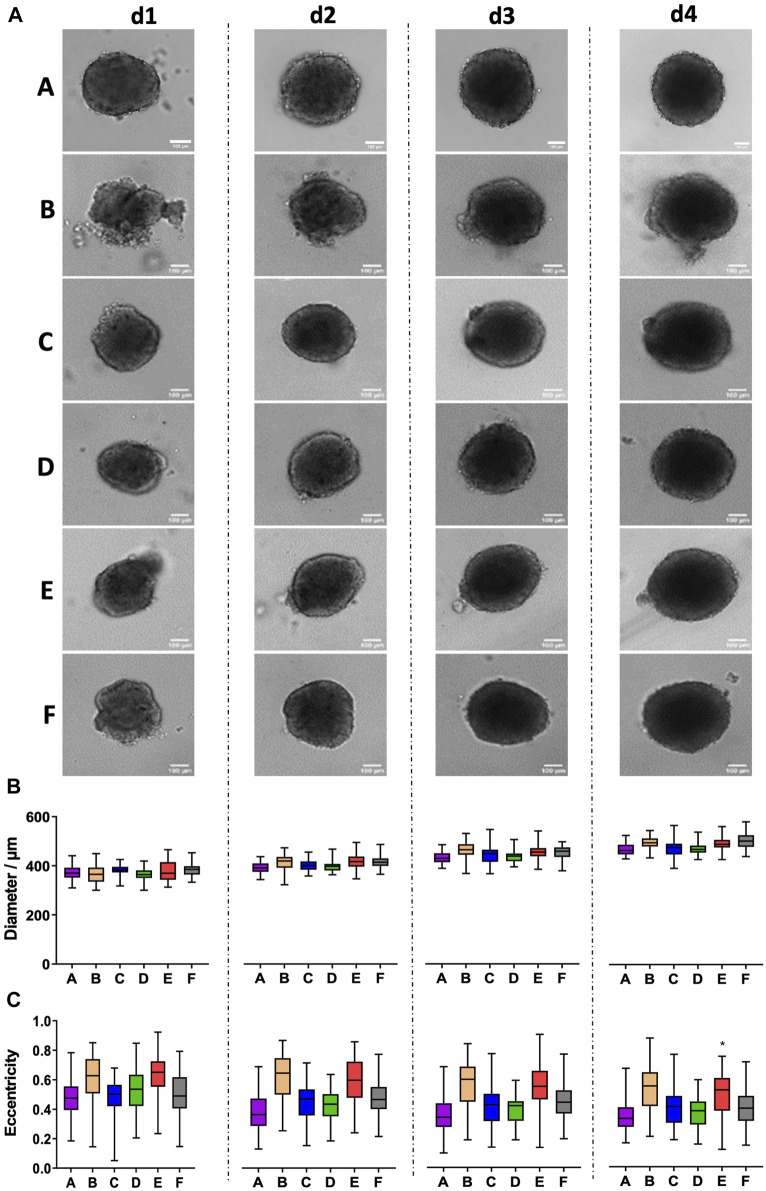
MDA-MB-231 breast cancer spheroids show satellite cell aggregates in plate types B and E. Freshly trypsinized MDA-MB-231 cells were seeded in the presence of 5 μg/mL collagen I into 96-well ULA plates, types A–F, at a density of 5,000 cells per well and then cultured for up to 4 days. Spheroid morphology was visualized daily using automated brightfield microscopy. **(A)** Representative micrographs showing individual spheroids from day 1 to day 4 (d1–d4) in plate types A–F. Scalebars, 100 μm. **(B, C)** Box-Whisker plots depicting spheroid diameters **(B)** or eccentricity **(C)** as a function of plate type A–F. Data are from three experiments with ≥ 24 spheroids per experiment (box: median, lower and upper quartiles; whiskers: Min to Max). Complete significance analysis, see Supplementary Figure S2. *, values significantly different compared to all other plate types for samples from the same day.

Next, we addressed the formation of spheroids using MDA-MB-231 breast cancer cells. Fitting to previous reports (Froehlich et al., 2016), MDA-MB-231 cells did not form spheroids alone but rather loose cell aggregates. Conversely, a consistent generation of stable and growing spheroids was observed for all plate types in the presence of collagen I upon seeding of 5,000 cells per well (Figure 2A). Quantitative analysis revealed that growth from day 1 to day 4 varied from 24% to 34% for different plate types (Figure 2B). Furthermore, in plate types B and E, the formation of smaller satellite cell aggregates that sometimes fused to the main spheroid was observed (Figure 2A). This led to significantly higher eccentricity values for these plate types, however with an inconsistent pattern of the culture days (Figure 2C).

### 3.3 HaCaT spheroid size and occurrence of loose cells and cell aggregates depends on plate type

**FIGURE 3:**
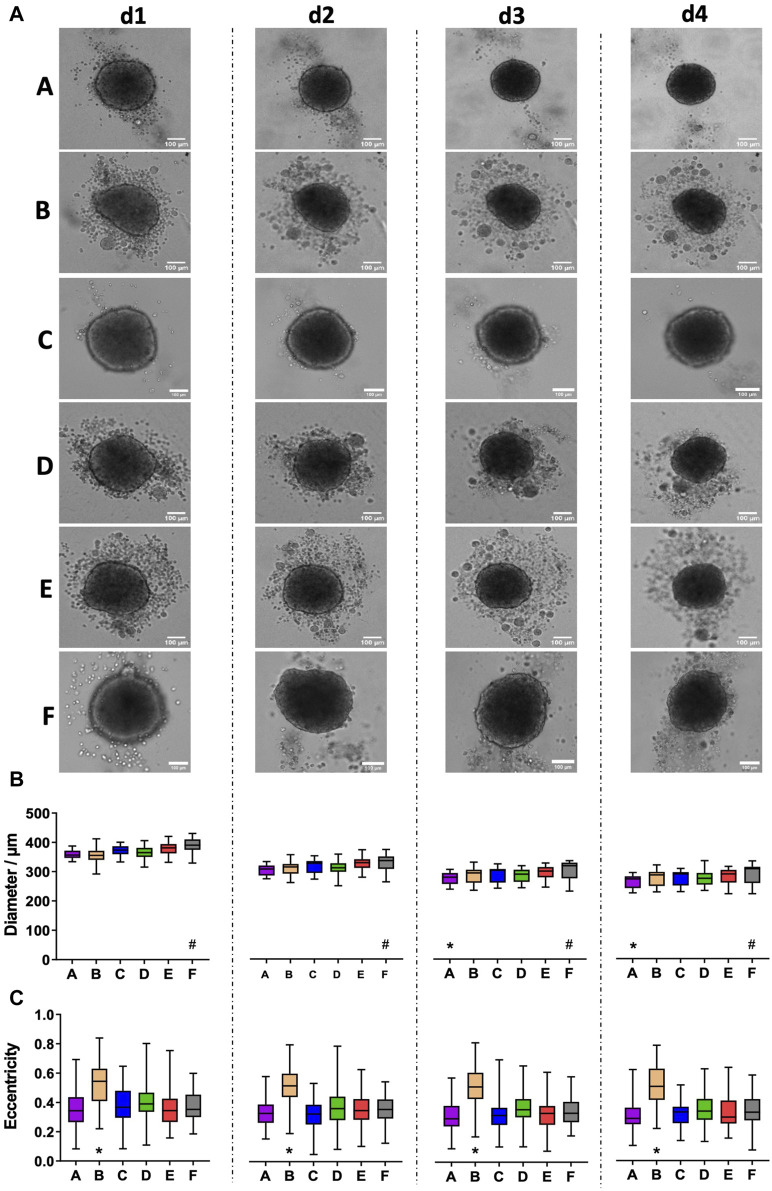
HaCaT keratinocyte spheroids vary in size and the occurrence of loose cells between different plate types. Freshly trypsinized HaCaT cells were seeded into 96-well ULA plates, types A-F, at a density of 5,000 cells per well and then cultured for up to 4 days. Spheroid morphology was visualized daily using automated brightfield microscopy. **(A)** Representative micrographs showing individual spheroids from day 1 to day 4 (d1–d4) in plate types A–F. Scalebars, 100 μm. **(B, C)** Box-Whisker plots depicting spheroid diameters **(B)** or eccentricity **(C)** as a function of plate type A–F. Data are from three experiments with ≥ 24 spheroids per experiment (box: median, lower and upper quartiles; whiskers: Min to Max). Complete significance analysis, see Supplementary Figure S3. * and #, values significantly different compared to all other plate types for samples from the same day.

**FIGURE 4:**
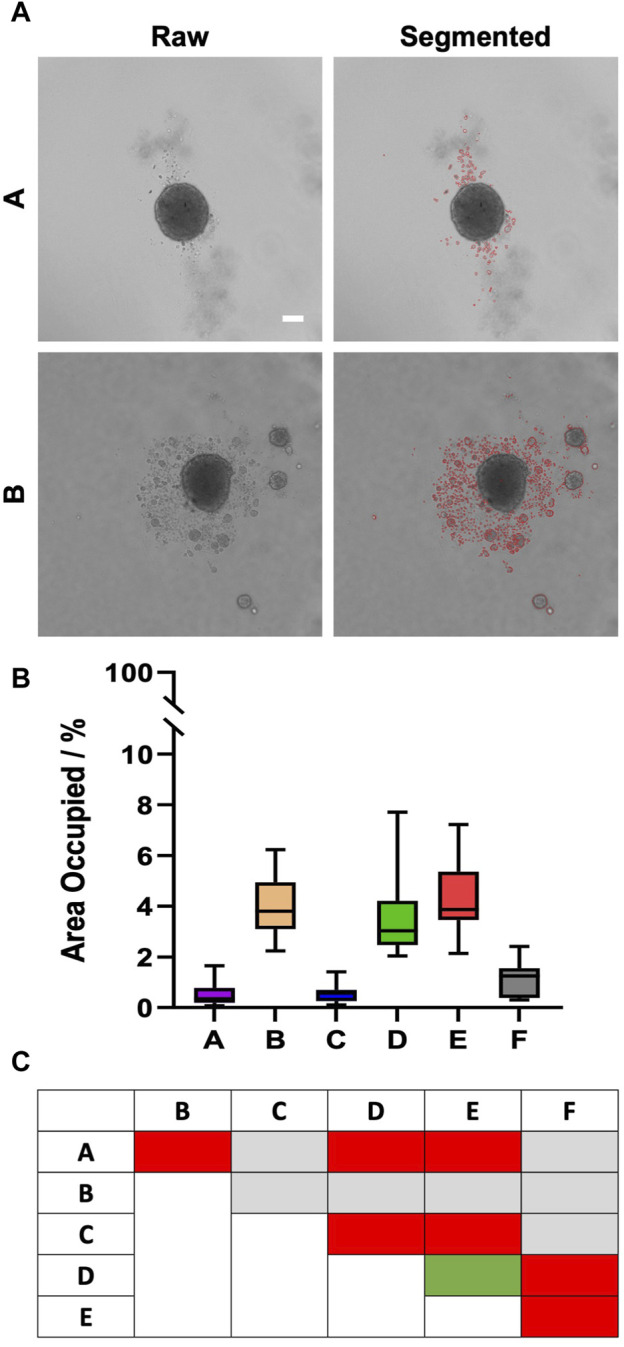
The occurrence of loose HaCaT cells and satellite cell aggregates is dependent on plate type. Freshly trypsinized HaCaT cells were seeded into 96-well ULA plates, types A-F, at a density of 5,000 cells per well and then cultured for 4 days. On day 4, spheroid morphology was visualized using automated brightfield microscopy. **(A)** Representative micrographs showing individual spheroids from plate types A and B, as indicated. The right panels depict regions outlined in red containing loose cells or satellite cell aggregates that were automatically segmented from raw images (left panels). Scalebar, 100 μm. **(B)** Box-Whisker plot exhibiting the percentage of the image area occupied by segmented regions (as exemplified in a) as a function of plate type A–F. Data are from three experiments with ≥ 12 spheroids per experiment (box: median, lower and upper quartiles; whiskers: Min to Max). **(C)** Table shows the statistical significance of Sidak multiple comparisons between the values of area occupied by loose cells and satellite mini-spheroids for plate types, as indicated. Colors stand for levels of significance: gray, n.s.; green, *p* ≤ 0.05; yellow, *p* ≤ 0.01; orange, *p* ≤ 0.001; red, *p* ≤ 0.0001.

In contrast to fibroblasts and MDA-MB231 cells, HaCaT spheroids, grown from 5,000 freshly trypsinized cells per well, showed a continued decrease in diameter (Figure 3A) from a value of roughly 350–380 µm on day 1 to approximately 270 µm on day 4 (Figure 3B). Within each day of observation, the plates with the largest spheroids on day 1 continued to harbor the biggest spheroids until day 4 (Figure 3B). From day 1–4, spheroids in plate type F were significantly larger than spheroids in all other types (Figure 3B, hashtags). On days 3 and 4, spheroids in plate type A were smaller than all the others (Figure 3B, asterisks). Furthermore, while plate types A, C, and F exhibited a clear surface surrounding the spheroids, numerous loose or non-attached cells and satellite cell aggregates were visible in all other plate types (Figure 3A). However, this did not correlate with spheroid size, as plates A and C showed small to medium-sized spheroids. Apart from the appearance of loose cells and satellite cell aggregates, the roundness of main spheroids varied between the different plate types. In particular, the spheroids raised in plate type B showed significantly higher eccentricity values than those of the other tested plates, meaning that their shape was less close to a perfect circle (Figure 3C, asterisks). Over the course of 4 days, eccentricity values did not change significantly for any given plate type (Figure 3C). Quantitative analysis confirmed that the area covered with loose cells and/or cell aggregates in the surroundings of the main spheroids was less than 0.5% in plate types A and C, while it was around 4% for types B, D, and E (Figures 4A, B). Plate type F assumed an intermediate position with 1.4% (Figure 4B). As shown in Figure 4C, the occurrence of loose cells and cell aggregates on day 4 was significantly different between plate types A, C, and F on one side, and types B, D, and E on the other side.

### 3.4 HT-29 spheroids exhibit increasing roundness during the growth phase and plate-type-dependent differences in size

**FIGURE 5:**
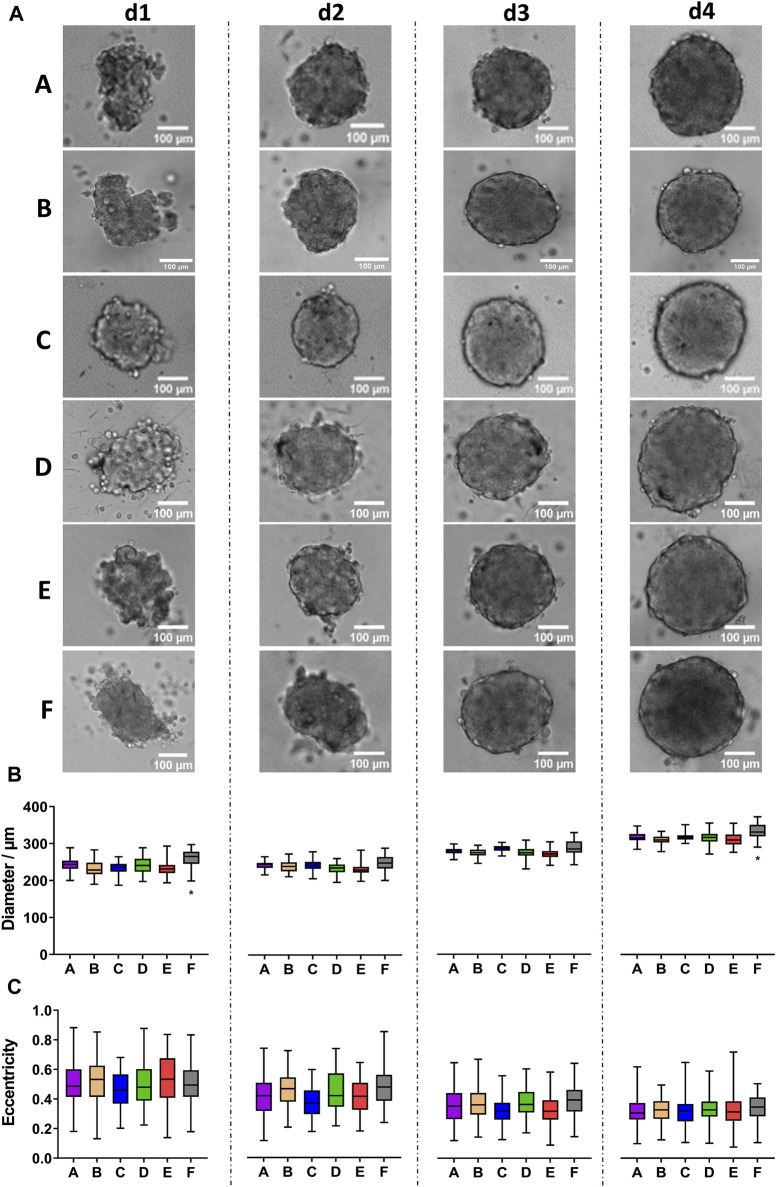
HT-29 colon cancer spheroids form robustly in all tested plate types. Freshly trypsinized HT-29 cells were seeded into 96-well ULA plates, types A-F, at a density of 500 cells per well and then cultured for up to 4 days. Spheroid morphology was visualized daily using automated brightfield microscopy. **(A)** Representative micrographs showing individual spheroids from day 1 to day 4 (d1–d4) in plate types A–F. Scalebars, 100 μm. **(B, C)** Box-Whisker plots depicting spheroid diameters **(B)** or eccentricity **(C)** as a function of plate type A-F. Data are from three experiments with ≥ 24 spheroids per experiment (box: median, lower and upper quartiles; whiskers: Min to Max). Complete significance analysis, see Supplementary Figure S4. *, values significantly different compared to all other plate types for samples from the same day.

Opposite to fibroblasts and keratinocytes, spheroids grown from 500 freshly trypsinized HT-29 colon cancer cells showed the typical, robust growth of highly proliferating cells (Figure 5A). While HT-29 cell aggregates were still mostly irregular during days 1 and 2 after seeding, they appeared as compact spheroids on day 3 and exhibited a round shape with a sharp border on day 4, regardless of the plate type. Quantitative analysis revealed an increase in the spheroid size of roughly 26% from day 1 to day 4 for all plate types (Figure 5B). However, on days 1 and 4, HT-29 spheroids from plate type F were consistently larger than those of all other types (Figure 5B, asterisk). Conversely, eccentricity values were largely alike between all plate types for a given day. Yet, for all plate types, eccentricity values decreased from approximately 0.5 to 0.3 (Figure 5C), corroborating the observed increase in roundness.

### 3.5 Cell proliferation, differentiation and YAP1 distribution in HaCaT spheroids varies with plate type

**FIGURE 6:**
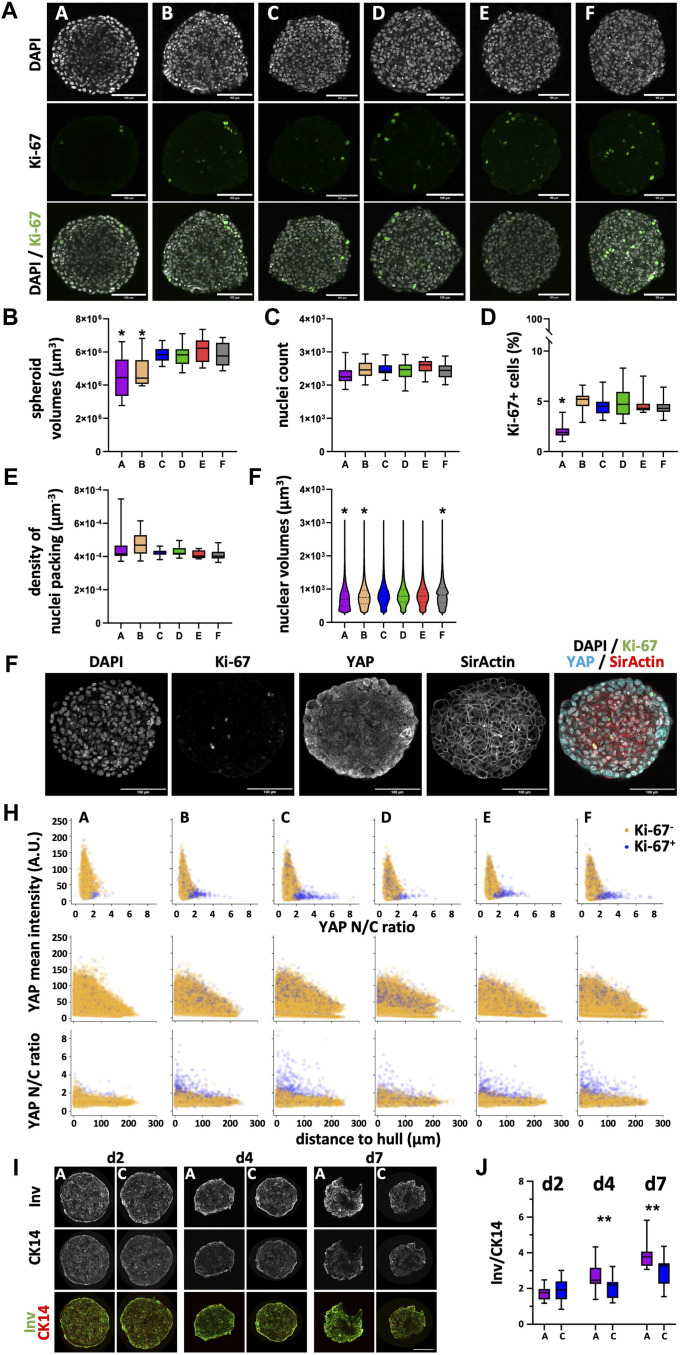
Differentiation and proliferation correlate with YAP1 in HaCaT spheroids and vary with the plate type. Freshly trypsinized HaCaT cells were seeded into 96-well ULA plates, types A-F, at a density of 5,000 cells per well and then cultured for 4 days **(A–H)** or for 2, 4, and 7 days **(I, J)**. Then, spheroids were fixed, cleared, and stained for nuclei and f-actin, for the proliferation marker, Ki-67, and YAP1 **(A–H)** or fixed, cryosliced and stained for the basal keratinocyte marker, CK14, and the differentiation marker, Involucrin (Inv, **(I, J)**. Wholemount confocal 3D microscopy and 3D-image segmentation **(A–H)** or cryosections and confocal microscopy **(I, J)** were performed. **(A)** Representative micrographs showing single optical sections through individual spheroid wholemounts at their largest circumference, from plate types as indicated. Upper panels, DAPI nuclear signals (gray); middle panels, Ki-67 immunofluorescence signals (green); lower panels, overlays. Scalebars, 100 μm. **(B–E)** Box-Whisker plots depicting the spheroid volumes **(B)**, the number of nuclei per spheroid **(C)**, the percentage of Ki-67+ nuclei of all nuclei **(D)**, and the density of nuclei packing within spheroids **(E)**. **(F)** Violin plot showing the size distribution of nuclear volumes in HaCaT spheroids as a function of plate type. Data in **(B–F)** are from three experiments with ≥ 24 spheroids per experiment (b-e, box: median, lower and upper quartiles; whiskers: Min to Max; f, median, lower and upper quartiles, and min to max are plotted). *, values significantly different compared to all plate types except A and B. Complete significance analysis, see Supplementary Figure S5. **(G)** Representative micrographs showing a single optical section through a spheroid wholemount at its largest circumference, from plate type F. Immunofluorescence signals of DAPI, gray; Ki-67, green; YAP1, cyan; f-actin, red. Scalebar, 100 µm. **(H)** Scatterplots showing values of all segmented cells (29,916–42,396 cells per plate type) for plate types A–F (indicated). Depicted are YAP1 mean intensity per cell as a function of YAP1 N/C ratio (upper row), YAP1 mean intensity per cell as a function of the cell’s distance to spheroid hull (middle row), YAP1 N/C ratio as a function of the cell’s distance to spheroid hull (lower row). Yellow and purple dots represent values of Ki-67- and Ki-67+ cells, respectively. **(I)** Representative confocal sum projections from cryoslices of spheroids harvested at day 2, 4, or 7 after seeding (indicated) from plate types A and C. In overlays, fluorescence signals of Involucrin, green; CK14, red. Scalebar, 100 µm. **(J)** Box plot depicting the Integrated Density ratio for Involucrin/CK14 fluorescence as a function of plate type and day of harvesting. Each data point is from 15 spheroids from three independent experiments. **, *p* ≤ 0.01.

Since spheroids grown from HaCaT and HT-29 cells showed overt and robust differences regarding spheroid size and since they were large enough to handle easily, a more in-depth analysis using wholemount imaging and 3D segmentation of immunostained spheroid wholemounts from all plate types was then performed for these two. To start with, HaCaT spheroids were fixed on day 4 and stained for nuclei, f-actin, the proliferation marker, Ki-67, and the proliferation- and differentiation-relevant transcriptional coregulator, YAP1. After optical tissue clearing, confocal 3D-microscopy yielded full-spheroid image data stacks with a good signal-to-noise ratio throughout all samples (see Figure 6A for representative optical sections through spheroid centers showing nuclei and Ki-67 signals). Automated AI-assisted segmentation of nuclei and Ki-67 was then performed. Consistent with the diameter analysis on live spheroids as shown in Figure 3, spheroids from plate type A showed the smallest volumes (Figure 6B; Supplementary Figure S5a). In addition, spheroids from plate type B were also significantly smaller than those from types C–F (Figure 6B; Supplementary Figure S5a). Now, while the number of nuclei per spheroid was comparable between all plate types (Figure 6C; Supplementary Figure S5b), the number of Ki-67+ cells was significantly lower in spheroids from plate type A (Figure 6D; Supplementary Figure S5c). Further, whereas the density of nuclei packing in spheroids was only slightly different between all plate types (Figure 6E; Supplementary Figure S5d), the distributions of nuclear volumes were significantly different between the plate types (Figure 6F; Supplementary Figure S5e). In detail, while the nuclei of spheroids from plate types C-E were very similarly distributed, spheroids from plate types A and B showed more nuclei with smaller volumes; this was particularly prominent for nuclei from plate type A (Figure 6F; Supplementary Figure S5e). Conversely, the size distribution of nuclei from plate type F exhibited a considerable fraction of large nuclei of around 1,000 μm^3^ (Figure 6F; Supplementary Figure S5e).

Previous work showed that YAP1 nuclear localization in adherent HaCaT cultures is dependent on cell density (Grannas et al., 2015), that YAP1 activity is primarily confined to the basal, proliferating keratinocyte layer of the epidermis (Rognoni and Walko, 2019), and that YAP1 activity is increased in activated keratinocytes during wound healing (Lertpatipanpong et al., 2023). This prompted us to investigate a potential correlation with the reduced amount of Ki-67+ HaCaT cells in spheroids from plate type A. Already at first glance, it was evident that YAP1 signal intensity increased towards the border of the spheroids (Figure 6G). Using the f-actin staining to identify the cell bodies and further algorithm pipelines to register the cell bodies to their corresponding nuclei, it became possible to automatically segment thousands of cells in these dense spheroids. This allowed us to determine single-cell values of YAP1 signal intensity as well as YAP1 N/C ratio, and to co-register these values to their Ki-67 status. As depicted in Figure 6H, this analysis confirmed the qualitative impression of an increased YAP1 signal intensity towards the spheroid border in all plate types (Figure 6H, middle row). Further, although Ki-67+ cells in plate types B-F exhibited a broad distribution of YAP1 mean intensity that largely overlapped with the values for Ki-67- cells, a subpopulation of Ki-67+ cells in these plate types exhibited a high YAP1 N/C ratio (Figure 6H, upper row, B–F) and this ratio was increasing towards the spheroid rim (Figure 6H, lower row, B–F). Notably, this subpopulation of Ki-67+ cells with high YAP1 N/C ratio was nearly absent in spheroids from plate type A (Figure 6H, upper row, A), suggesting that these might be the ones missing in the total Ki-67+ cell counts (Figure 6D).

Since keratinocyte differentiation is normally preceded by exit from the cell cycle (Rognoni and Walko, 2019), we hypothesized that the reduction in proliferating cells observed in spheroids of plate type A might be due to enhanced HaCaT cell differentiation. Therefore, HaCaT spheroids were cultured in plate types A and C, harvested after 2, 4, and 7 days after seeding, cryosectioned, and stained for the basal keratinocyte marker, cytokeratin 14 (CK14), and for Involucrin, a marker of more differentiated cells. Qualitatively, this showed the expected decrease in diameter from early to late time points for spheroids from both plate types (Figure 6I). While CK14 was evenly distributed throughout the spheroid diameters, Involucrin was more concentrated towards the spheroid borders (Figure 6I). Quantitative analysis of the Involucrin/CK14 ratio showed that although spheroids from both plate types exhibited an increase of Involucrin/CK14 ratio with time, arguing for an ongoing differentiation process. Yet, the rise in Involucrin/CK14 ratio was more pronounced in the spheroids from plate type A (Figure 6J). In summary, these data were consistent with a plate-type-dependent variation of YAP1 expression and YAP1 N/C ratio and with a concomitant antagonistic regulation of cell proliferation and differentiation.

### 3.6 Neither cell proliferation nor YAP1 distribution varies in HT-29 spheroids with plate type

**FIGURE 7:**
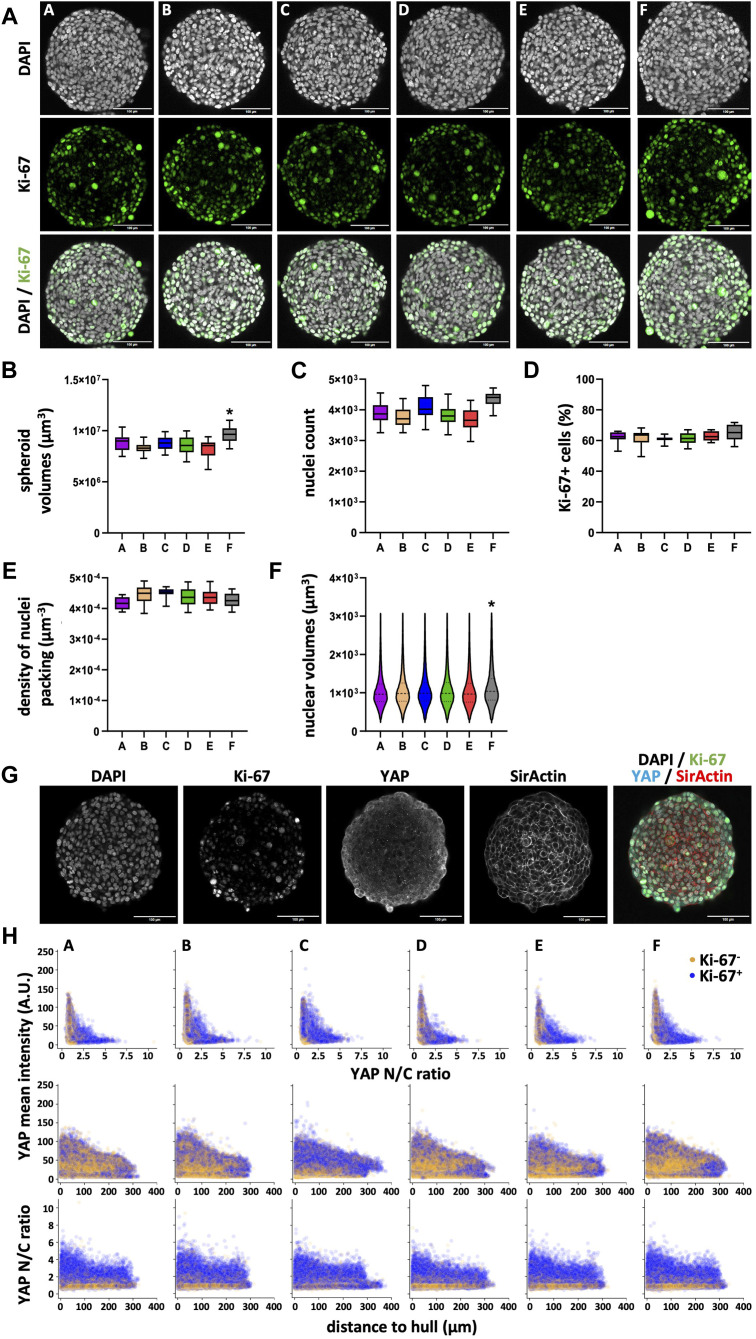
Nuclear counts and nuclear volume distribution vary in HT-29 spheroids depending on the plate type. Freshly trypsinized HT-29 cells were seeded into 96-well ULA plates, types A–F, at a density of 500 cells per well and then cultured for 4 days. On day 4, spheroids were fixed, cleared, and stained for nuclei and f-actin, for the proliferation marker, Ki-67, and YAP1. Wholemount confocal 3D microscopy and 3D-image segmentation were performed. **(A)** Representative micrographs showing single optical sections through individual spheroids at their largest circumference, from plate types as indicated. Upper panels, DAPI nuclear signals (gray); middle panels, Ki-67 immunofluorescence signals (green); lower panels, overlays. Scalebars, 100 μm. **(B–E)** Box-Whisker plots depicting the spheroid volumes **(B)**, the number of nuclei per spheroid **(C)**, the percentage of Ki-67+ nuclei of all nuclei **(D)**, and the density of nuclei packing within spheroids **(E)**. **(F)** Violin plot showing the size distribution of nuclear volumes in HT-29 spheroids as a function of plate type. Data in **(B–F)** are from three experiments with ≥ 24 spheroids per experiment (b–e, box: median, lower and upper quartiles; whiskers: Min to Max; f, median, lower and upper quartiles, and min to max are plotted). *, values significantly different compared to all other plate types. Complete significance analysis, see Supplementary Figure S6. **(G)** Representative micrographs showing a single optical section through a spheroid wholemount at its largest circumference, from plate type **(F)**. Immunofluorescence signals of DAPI, gray; Ki-67, green; YAP1, cyan; f-actin, red. Scalebar, 100 µm. **(H)** Scatterplots showing values of all segmented cells (59,337–74,649 cells per plate type) for plate types A–F (indicated). Depicted are YAP1 mean intensity per cell as a function of YAP1 N/C ratio (upper row), YAP1 mean intensity per cell as a function of the cell’s distance to spheroid hull (middle row), YAP1 N/C ratio as a function of the cell’s distance to spheroid hull (lower row). Yellow and purple dots represent values of Ki-67- and Ki-67+ cells, respectively.

Similar to the HaCaT cells, to investigate the diameter differences observed between HT-29 spheroids from plate type F vs. types A-E, we again performed the wholemount confocal analysis (see Figure 7A for representative images). Quantitative determination of spheroid volumes (Figure 7B) confirmed the diameter measurements in live cells (Figure 5B). Indeed, spheroids from plate type F had larger volumes compared to all other types (Figure 7B; Supplementary Figure S6a). This was also reflected by higher nuclei counts in plate F spheroids (Figure 7C; Supplementary Figure S6b, ns. comparing C and F). Conversely, the number of proliferating cells (Figure 7D; Supplementary Figure S6c) and the density of nuclei packing (Figure 7E; Supplementary Figure S6d) were rather similar between all plate types. Finally, nuclei from plate type F spheroids showed a different size distribution compared to those of all other plate types (Figure 7F; Supplementary Figure S6e). In particular, nuclei with a larger volume (>1,000 μm^3^) were more abundant in these spheroids. Concerning YAP1, both mean intensity and N/C ratio showed a gradient from the center to the border of spheroids (Figures 7G, H). However, at difference to HaCaT spheroids, YAP1 mean intensity of Ki-67+ cells largely overlapped with that of Ki-67- cells (Figure 7H).

## 4 Discussion

Single-cell analyses are powerful approaches to reveal the complexity of cellular interactions in 3D-cell cultures. Among these technologies, the 3D-segmentation of fluorescence marker-labeled image data is particularly potent in addressing the spatial vicinity of convergent or divergent processes at a single-cell level. While AI-assisted approaches to determine nuclear number or cell shape have recently improved a lot (Dunn et al., 2019; Stringer et al., 2021; von Chamier et al., 2021), these studies have often remained conceptual, i.e., applied to a limited data set or to small 3D-cultures of few hundred cells (Pelt, 2020; Wang et al., 2022; Han et al., 2023). Furthermore, technologies to simultaneously quantify fluorescence data from more than one subcellular compartment at the single-cell level are still in their infancies (Gilles et al., 2017; Khushi et al., 2017; Tosi et al., 2020; Dubos et al., 2022). Here, we developed a procedure composed of nuclear and cell-membrane segmentation followed by subsequent cell-identity fusion to determine the N/C-ratio of the transcriptional coregulator, YAP1, the expression of the proliferation marker, Ki-67, and a series of other morphometric indicators at a single-cell level in large spheroids. Systematically applied to spheroids made from different cell lines and in distinct types of ULA-plates, this line of action revealed a correlation of YAP1 N/C-ratio with cell proliferation and differentiation in keratinocyte spheroids. Furthermore, the study served as a systematic test, if and to what extent different cell culture surfaces affect the outcome of spheroid formation and growth. For six different plate types, the present study systematically addressed morphological characteristics of spheroids prepared from four distinct cell types, over the course of 4 days after seeding. Although this revealed that, in principle, all tested plate types succeeded in reliably producing spheroids of all cell lines under investigation, significant differences in spheroid size and roundness, as well as cell number and proliferation were observed in a cell-line-dependent manner. This highlights that the choice of plate type may affect experimental outcomes and that consistency in the use of a specific plate type is critical during experimental campaigns.

As expected, growth was static or negative for non-neoplastic fibroblast cells and keratinocytes (Figures 1, 3), respectively, while it was positive for breast and colon cancer cells (Figures 2, 5). These patterns were similar between all six ULA-plate types tested. However, spheroids of all cell types raised in plate type F were consistently larger than in all other plate types and those from plate types A (for HaCaT and MDA-MB-231) and B (for CCD-1137Sk and HaCaT) were smaller than spheroids from the other plate types. A more in-depth confocal wholemount analysis for HaCaT and HT-29 cells revealed that the reasons for these discrepancies might be different ones, depending on the cell type. Indeed, for HaCaT cells, the overall cell number was similar when comparing spheroids from all six plate types (Figure 6C), while it was different in the case of HT-29 cells (Figure 7C), at least comparing plate type F with A, B, D, and E. Thus, while for HT-29 cells a higher cell number might explain the larger spheroids in plate type F, this scenario was unlikely in HaCaT spheroids. Notably, HaCaT spheroids of plate types A and B were smaller on d4 according to confocal data (Figure 6A), but only those of plate type A were also smaller on d4 according to brightfield (Figures 3A,B). Currently, it is unclear, where this inconsistency could arise from. However, the smaller size of plate type A spheroids was reflected by a slightly lower nuclei count (ns, Figure 6C), a lower number of Ki-67+ cells (Figure 6D), and a lack of cells that were Ki-67+ and simultaneously showed a high YAP N/C ratio (Figure 6H). Furthermore, the smaller size of plate type A and B spheroids was likely reflected by a trend towards a higher density of nuclei packing (ns, Figure 6E) and significantly smaller nuclear volumes (Figure 6F).

In general, nuclear size and shape are determined by several factors, including alterations in nuclear transport, cell cycle regulation, genome packaging, genome activity, cellular physiology, and/or cell viability (Manda et al., 2023). In the specific case of HaCaT keratinocytes, nuclear morphology varied according to their differentiation status (Gniadecki et al., 2001), at least in 2D cultures, where incubation in high Ca^2+^ levels leads to an increased expression of differentiated keratinocyte markers (Deyrieux and Wilson, 2007). In spheroids, HaCaT cells were also found to exhibit a stratification with more differentiated cells towards the spheroid rim (Klicks et al., 2019). Notably, in that study, HaCaT differentiation was lost upon coculture with melanoma cells and regained after cytostatic-induced killing of melanoma cells. Furthermore, it showed a cytostatic-mediated upregulation of ABCB5, a xenobiotic transporter involved in drug resistance (Klicks et al., 2019). On the other hand, epidermal spheroid models fall short of mimicking human skin at least in two major points. First, while cell proliferation in human epidermis is limited to the stratum basale, our HaCaT spheroids showed Ki-67+ cells throughout the spheroid width. Second, human epidermis normally exhibits a defined organization ranging from basal over spinous and granular to cornified layers (Lintzeri et al., 2022). These layers are characterized by different functions, including cell proliferation, production and secretion of shielding proteins and lipids, barrier function, and apoptotic cell death to form the cornified layer. Compared to this, the HaCaT spheroids showed only a reduced complexity. We observed production and enrichment of Involucrin at the rim of our spheroids (Figure 6I). Involucrin is normally expressed by cells of spinous and granular layers, and then secreted to participate together with apoptotic cells in the formation of the cornified layer (Eckert et al., 2004). Yet, our spheroids did not feature a *bona fide* cornified layer, which even in full-thickness skin equivalent models necessitates an air lifting procedure to form. In addition, the stratification of CK14 and Involucrin was not nearly as pronounced as in human skin or in full-thickness models. Thus, spheroid-based epidermal models are inferior with respect to classical full-thickness human skin equivalents in terms of differentiation capacity, distribution of proliferative cells, and predictive power (Jahn et al., 2024; Kurzyk et al., 2024). Nonetheless, they display some critical features of keratinocyte physiology. In combination with their fast and easy production, keratinocyte spheroids can therefore serve as simplified models with intermediate complexity between 2D-cell culture and skin equivalent models. As mentioned, during epidermal differentiation, keratinocytes become postmitotic before they go into apoptosis (Yin et al., 2023). Thus, as for many epithelial cell types, there is an inverse correlation between proliferation and differentiation of epidermal keratinocytes. The Hippo signaling pathway with its downstream effectors, YAP and TAZ, is particularly relevant to control epidermal homeostasis. Indeed, active, i.e., nuclear YAP is normally present only in keratinocytes showing proliferative activity, namely in the basal layer of the epidermis (Rognoni and Walko, 2019). Furthermore, it is upregulated in states of enhanced growth, i.e., upon wound healing and in epidermal cancers (Rognoni and Walko, 2019). This was corroborated by our findings that HaCaT spheroids from plate type A showed a lower amount of Ki-67+ proliferating cells (Figure 6D; Supplementary Figure S7), a higher propensity to maturate (Figures 6I,J) and a lack of Ki-67+ cells exhibiting a high YAP1 N/C ratio (Figure 6H). At present, it is unclear if these findings are a coincidence or confounded by the non-physiological distribution of proliferating cells in the spheroid model. Also, given that YAP1 intensity as well as YAP1 N/C ratio were strongly increasing from center to border of the HaCaT spheroids in all plate types (Figure 6H), more general effects could be relevant. For example, YAP1 was described to be important for negative durotaxis in melanoma cells (Huang et al., 2022) and to be less present in the nuclei of well-polarized epithelia (van Soldt and Cardoso, 2020). Although experimental data for this are missing, the YAP distribution results might suggest a stiffness gradient from HaCaT spheroid border to center and that the cells of the spheroid rim lacked well-developed epithelial polarization. Further work is needed to pinpoint the causality and underlying mechanisms.

Regarding the HT-29 colon cancer cells, only spheroids grown in plate type F were significantly different in size from those raised in all other plate types (Figures 5, 7B). As mentioned earlier, this could be likely explained by the higher cell numbers counted in HT-29 spheroids from plate type F (Figure 7C). However, in these samples, also the nuclear volumes were significantly larger than in those from plate types A-E (Figure 7F). Currently, it is unclear if this contributed to the observed differences in spheroid size. Moreover, the underlying reasons for the altered nuclear volume are difficult to explain. Indeed, cell proliferation as evaluated by the fraction of Ki-67+ cells as well as the analysis of YAP1 distribution and N/C ratio were inconspicuous (Figures 7D, H; Supplementary Figure S7). Further options would be differences in apoptosis, pressure (Khavari and Ehrlicher, 2019), or stiffness.

Finally, this study also revealed plate-type dependent differences in spheroid roundness and the presence of loose cells or cell aggregates. These effects were primarily observed in CCD-1137Sk (Figure 1), HaCaT (Figures 3, 4), and MDA-MB-231 cells (Figure 2). First, that loose cells or cell aggregates were found might have different reasons, depending also on the cell type. Given that spheroid formation as such is primarily induced by depriving cells of an adherent surface, therefore forcing them to interact with each other, the access to extracellular matrix and to cell-cell contacts as well as the possibility to achieve cellular polarity are altered as compared to 2D cultures (and organoids, see below). This, in turn, might be tolerated to a different degree depending on the cell’s inherent program. While fibroblasts are usually sparsely distributed and primarily contact extracellular matrix, epithelial cells normally interact with the extracellular matrix mainly on their basal side and are linked via adhesion junctions and often also tight junctions at their lateral sides (Wang et al., 2021). Fittingly, while spheroids from fibroblasts were rather loosely arranged and irregular in shape (Figure 1), HaCaT spheroids were more compact and showed a regular round appearance (Figure 3). Cancer cells, depending on their origin and metastatic potential, often undergo epithelial-mesenchymal transition, whereby cell-cell interactions are reduced at the expense of cell-matrix interactions (Vandyck et al., 2021). Consistently, in the present study, HT-29 cells, which in 2D form epithelial-like cell clusters (Keller et al., 2020) and which are known to express E-cadherin (Al-toub et al., 2015), easily formed smooth and round spheroids (Figure 5), while the highly metastatic MDA-MB-231 cells, lacking E-cadherin (Al-toub et al., 2015), needed the addition of collagen to form spheroids (Figure 2). Furthermore, even within a given cell type there might be a variability of tolerance towards delayed cell-cell/cell-matrix contact formation and subsequent anoikis. For example, while the expression level of protein kinase N1 was critical for cell aggregation and spheroid formation of mesenchymal cells (Mehruba et al., 2020), the abundance of epidermal growth factor receptor determined the spheroid-forming ability of normal human keratinocytes (Woappi et al., 2020). Since details on the surfaces of ULA plates are usually not disclosed, it is unclear what induced the differences detected in the present study. However, it has become clear that the selection of a specific plate type can have profound consequences on major morphological and cell biological parameters. This warrants a careful consideration upfront to the execution of experimental campaigns and supports the need to implement different plate types depending on the nature of the investigation.

## 5 Outlook–spheroids vs. organoids and their automated analysis

While our study focused on spheroids, principal tools of the study, including the optical tissue clearing, 3D-fluorescence staining and microscopy, as well as 3D-image analysis and quantitative evaluation are transferable to organoids as the physiologically more relevant models. Future studies will benefit from applying the presented measurement pipeline for comparing spheroid and organoid models to better understand differences regarding the expression of key markers and pathway activities, thereby informing their use in drug sensitivity assays.

Spheroids typically refer to aggregates of one or more cell types that form a spherical cell culture, often driven by non-adhesive culture conditions, while organoids are derived from stem cells and are capable of self-organizing into structures that mimic critical features of architecture and functionality of actual organs (Fatehullah et al., 2016). Due to their ease of production, low cost, high reproducibility, and the formation of cell-cell as well as cell-matrix contacts, and due to the presence of gradients for oxygen, nutrients, waste products, and drugs, spheroids are useful models for drug screens with a higher physiological relevance than with classical 2D-cultures (Bloise et al., 2024). In particular, upon coculture of differently aggressive tumor cell lines or with fibroblasts, immune cells, etc., spheroids can also mimic tumor heterogeneity and stromal cell-cell interactions (Fröhlich, 2023; Bloise et al., 2024). Furthermore, upon use of hydrogels or matrix components, effects of extracellular matrix and stiffness on cellular behavior can be assessed (Fröhlich, 2023; Bloise et al., 2024). However, since spheroids are typically made of cell lines and generated by forcing cell-cell interaction through depriving cells of an adherent surface, spheroids are weak in addressing personalized medicine and they usually exhibit only modest levels of differentiation.

Organoids, due to their stem cell origin and self-organizing capabilities, often show a higher degree of cellular differentiation and tissue-like organization compared to spheroids (Crespo et al., 2017; Campaner et al., 2020; Ray, 2020; Mohan et al., 2021; Bhattacharya et al., 2023; Yang et al., 2024). Organoids can recapitulate the spatial orientation and cell-type diversity of their tissue of origin, such as intestinal crypt-villus structures (Sato et al., 2009) or the complex layering of the brain cortex (Lancaster et al., 2013; Lancaster and Knoblich, 2014). This level of organization is not reached in spheroids, which may result in differences in the expression of differentiation markers. For example, intestinal organoids express differentiated cell markers like Lgr5 and mucin-2, whereas spheroids may show a less distinct differentiation profile (Sato et al., 2009; Munro et al., 2023). The Hippo signaling pathway, and particularly the activity of YAP1, is closely linked to cell density, mechanical cues, and the architecture of the cell culture (Panciera et al., 2017). In organoids, YAP1 localization and activity can vary significantly between different regions of the structure, reflecting the complex tissue architecture and mechanical environment (Bhattacharya et al., 2023). For instance, YAP1 is typically localized to the nuclei in proliferative zones and excluded from nuclei in differentiated regions of organoids (Natekar et al., 2021). This spatial regulation of YAP1 may be less pronounced in spheroids, where the mechanical and structural cues are more homogenous. The differentiation state and cellular architecture of 3D cultures can profoundly influence drug response. Organoids, with their higher degree of differentiation and tissue-like organization, often exhibit drug responses that are more predictive of *in vivo* outcomes compared to spheroids (Vlachogiannis et al., 2018; LeBlanc et al., 2021). This is particularly important for drug sensitivity assays where the cellular context and microenvironment can affect the efficacy of therapeutic agents. For example, a special type of colon cancer organoids was needed to identify a quiescent group of cancer cells, so called persister cells, and their dependence on the extracellular matrix and YAP1 upregulation (Ohta et al., 2022). In summary, while organoids are considered to be superior to spheroids in terms of physiological relevance, spheroids are rapidly produced in high numbers and low technical and biological variability, rendering both of them interesting tools for high-content/high-throughput analyses and industrial drug prescreens. Future work will tell, if a combination of 2D, spheroid, and organoid approaches will lead to a rise in efficiency, reliability, and predictive power of biomedical drug screen campaigns. On that way, continuously improved means of 3D-image analysis and combinatorial multiparametric analyses will increasingly deepen our insights into the correlation between morphology and function, between cell type and drug effect in complex spheroid and organoid 3D-cultures. Along these lines, the present work has contributed new tools to assess subcellular distribution of markers and morphometric features by instance mapping.

## Data Availability

The raw data supporting the conclusions of this article will be made available by the authors, without undue reservation.
