# Supplementary figures and images for: A multiparametric analysis including single-cell and subcellular feature assessment reveals differential behavior of spheroid cultures on distinct ultra-low attachment plate types
Source: Front Bioeng Biotechnol. 2024 Aug 2;12:1422235. doi: 10.3389/fbioe.2024.1422235 (PMC11327450; doi:10.3389/fbioe.2024.1422235)

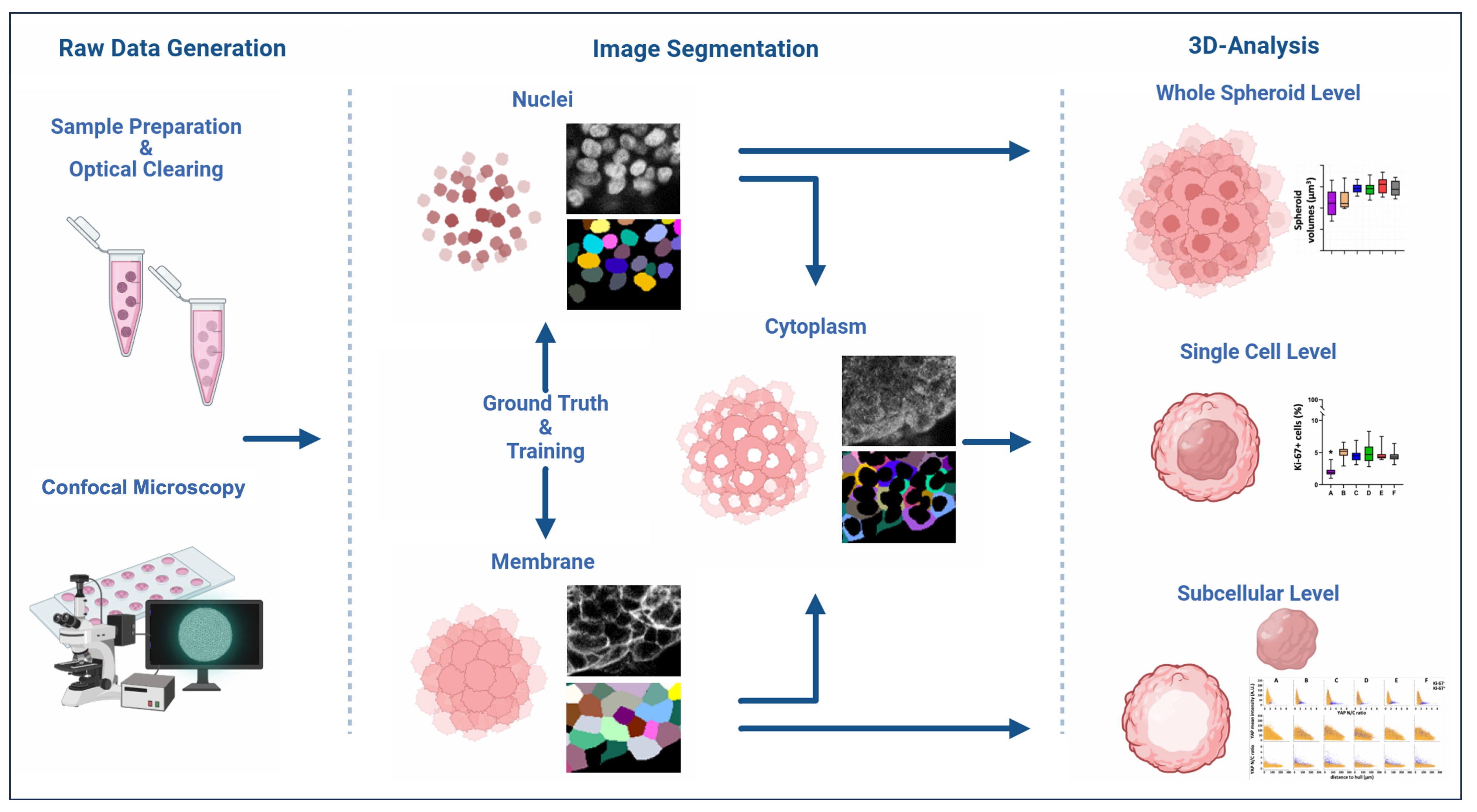

Supplement: Supplementary file 1 [file Image1.JPEG]
