# Supplementary material for: A multiparametric analysis including single-cell and subcellular feature assessment reveals differential behavior of spheroid cultures on distinct ultra-low attachment plate types
Source: Front Bioeng Biotechnol. 2024 Aug 2;12:1422235. doi: 10.3389/fbioe.2024.1422235 (PMC11327450; doi:10.3389/fbioe.2024.1422235)
Supplement: Supplementary file 2 [file DataSheet1.docx]

Supplementary Material to Manuscript:

Vitacolonna et al.: A Multiparametric Analysis Including Single-Cell and Subcellular Feature Assessment Reveals Differential Behavior of Spheroid Cultures on Distinct Ultra-Low Attachment Plates Types

## Supplementary Figure 1

**a)**

|  | B | | | | C | | | | D | | | | E | | | | F | | | |
| --- | --- | --- | --- | --- | --- | --- | --- | --- | --- | --- | --- | --- | --- | --- | --- | --- | --- | --- | --- | --- |
|  | d1 | d2 | d3 | d4 | d1 | d2 | d3 | d4 | d1 | d2 | d3 | d4 | d1 | d2 | d3 | d4 | d1 | d2 | d3 | d4 |
| A |  |  |  |  |  |  |  |  |  |  |  |  |  |  |  |  |  |  |  |  |
| B |  | | | |  |  |  |  |  |  |  |  |  |  |  |  |  |  |  |  |
| C |  |  |  |  |  | | | |  |  |  |  |  |  |  |  |  |  |  |  |
| D |  |  |  |  |  |  |  |  |  | | | |  |  |  |  |  |  |  |  |
| E |  |  |  |  |  |  |  |  |  |  |  |  |  | | | |  |  |  |  |

**b)**

|  | B | | | | C | | | | D | | | | E | | | | F | | | |
| --- | --- | --- | --- | --- | --- | --- | --- | --- | --- | --- | --- | --- | --- | --- | --- | --- | --- | --- | --- | --- |
|  | d1 | d2 | d3 | d4 | d1 | d2 | d3 | d4 | d1 | d2 | d3 | d4 | d1 | d2 | d3 | d4 | d1 | d2 | d3 | d4 |
| A |  |  |  |  |  |  |  |  |  |  |  |  |  |  |  |  |  |  |  |  |
| B |  | | | |  |  |  |  |  |  |  |  |  |  |  |  |  |  |  |  |
| C |  |  |  |  |  | | | |  |  |  |  |  |  |  |  |  |  |  |  |
| D |  |  |  |  |  |  |  |  |  | | | |  |  |  |  |  |  |  |  |
| E |  |  |  |  |  |  |  |  |  |  |  |  |  | | | |  |  |  |  |

**Figure S1: CCD-1137Sk spheroids vary primarily in size between different plate types.** Tables display statistical significance of Sidak multiple comparisons between values of a) spheroid diameter and b) spheroid eccentricity for plate types and days in culture (d1-d4) as indicated, experimental data as depicted in Figure 1. Colors indicate levels of significance: gray, n.s.; green, p ≤ 0.05; yellow, p ≤ 0.01; orange, p ≤ 0.001; red, p ≤ 0.0001.

## Supplementary Figure 2

**a)**

|  | B | | | | C | | | | D | | | | E | | | | F | | | |
| --- | --- | --- | --- | --- | --- | --- | --- | --- | --- | --- | --- | --- | --- | --- | --- | --- | --- | --- | --- | --- |
|  | d1 | d2 | d3 | d4 | d1 | d2 | d3 | d4 | d1 | d2 | d3 | d4 | d1 | d2 | d3 | d4 | d1 | d2 | d3 | d4 |
| A |  |  |  |  |  |  |  |  |  |  |  |  |  |  |  |  |  |  |  |  |
| B |  | | | |  |  |  |  |  |  |  |  |  |  |  |  |  |  |  |  |
| C |  |  |  |  |  | | | |  |  |  |  |  |  |  |  |  |  |  |  |
| D |  |  |  |  |  |  |  |  |  | | | |  |  |  |  |  |  |  |  |
| E |  |  |  |  |  |  |  |  |  |  |  |  |  | | | |  |  |  |  |

**b)**

|  | B | | | | C | | | | D | | | | E | | | | F | | | |
| --- | --- | --- | --- | --- | --- | --- | --- | --- | --- | --- | --- | --- | --- | --- | --- | --- | --- | --- | --- | --- |
|  | d1 | d2 | d3 | d4 | d1 | d2 | d3 | d4 | d1 | d2 | d3 | d4 | d1 | d2 | d3 | d4 | d1 | d2 | d3 | d4 |
| A |  |  |  |  |  |  |  |  |  |  |  |  |  |  |  |  |  |  |  |  |
| B |  | | | |  |  |  |  |  |  |  |  |  |  |  |  |  |  |  |  |
| C |  |  |  |  |  | | | |  |  |  |  |  |  |  |  |  |  |  |  |
| D |  |  |  |  |  |  |  |  |  | | | |  |  |  |  |  |  |  |  |
| E |  |  |  |  |  |  |  |  |  |  |  |  |  | | | |  |  |  |  |

**Figure S2: MDA-MB-231 spheroid generation is variable between different plate types.** Tables display statistical significance of Sidak multiple comparisons between values of a) spheroid diameter and b) spheroid eccentricity for plate types and days in culture (d1-d4) as indicated, experimental data as depicted in Figure 2. Colors indicate levels of significance: gray, n.s.; green, p ≤ 0.05; yellow, p ≤ 0.01; orange, p ≤ 0.001; red, p ≤ 0.0001.

## Supplementary Figure 3

**a)**

|  | B | | | | C | | | | D | | | | E | | | | F | | | |
| --- | --- | --- | --- | --- | --- | --- | --- | --- | --- | --- | --- | --- | --- | --- | --- | --- | --- | --- | --- | --- |
|  | d1 | d2 | d3 | d4 | d1 | d2 | d3 | d4 | d1 | d2 | d3 | d4 | d1 | d2 | d3 | d4 | d1 | d2 | d3 | d4 |
| A |  |  |  |  |  |  |  |  |  |  |  |  |  |  |  |  |  |  |  |  |
| B |  | | | |  |  |  |  |  |  |  |  |  |  |  |  |  |  |  |  |
| C |  |  |  |  |  | | | |  |  |  |  |  |  |  |  |  |  |  |  |
| D |  |  |  |  |  |  |  |  |  | | | |  |  |  |  |  |  |  |  |
| E |  |  |  |  |  |  |  |  |  |  |  |  |  | | | |  |  |  |  |

**b)**

|  | B | | | | C | | | | D | | | | E | | | | F | | | |
| --- | --- | --- | --- | --- | --- | --- | --- | --- | --- | --- | --- | --- | --- | --- | --- | --- | --- | --- | --- | --- |
|  | d1 | d2 | d3 | d4 | d1 | d2 | d3 | d4 | d1 | d2 | d3 | d4 | d1 | d2 | d3 | d4 | d1 | d2 | d3 | d4 |
| A |  |  |  |  |  |  |  |  |  |  |  |  |  |  |  |  |  |  |  |  |
| B |  | | | |  |  |  |  |  |  |  |  |  |  |  |  |  |  |  |  |
| C |  |  |  |  |  | | | |  |  |  |  |  |  |  |  |  |  |  |  |
| D |  |  |  |  |  |  |  |  |  | | | |  |  |  |  |  |  |  |  |
| E |  |  |  |  |  |  |  |  |  |  |  |  |  | | | |  |  |  |  |

**Figure S3: HaCaT spheroid size and eccentricity depend on plate type.** Tables display statistical significance of Sidak multiple comparisons between values of a) spheroid diameter and b) spheroid eccentricity for plate types and days in culture (d1-d4) as indicated, experimental data as depicted in Figure 3. Colors indicate levels of significance: gray, n.s.; green, p ≤ 0.05; yellow, p ≤ 0.01; orange, p ≤ 0.001; red, p ≤ 0.0001.

## Supplementary Figure 4

**a)**

|  | B | | | | C | | | | D | | | | E | | | | F | | | |
| --- | --- | --- | --- | --- | --- | --- | --- | --- | --- | --- | --- | --- | --- | --- | --- | --- | --- | --- | --- | --- |
|  | d1 | d2 | d3 | d4 | d1 | d2 | d3 | d4 | d1 | d2 | d3 | d4 | d1 | d2 | d3 | d4 | d1 | d2 | d3 | d4 |
| A |  |  |  |  |  |  |  |  |  |  |  |  |  |  |  |  |  |  |  |  |
| B |  | | | |  |  |  |  |  |  |  |  |  |  |  |  |  |  |  |  |
| C |  |  |  |  |  | | | |  |  |  |  |  |  |  |  |  |  |  |  |
| D |  |  |  |  |  |  |  |  |  | | | |  |  |  |  |  |  |  |  |
| E |  |  |  |  |  |  |  |  |  |  |  |  |  | | | |  |  |  |  |

**b)**

|  | B | | | | C | | | | D | | | | E | | | | F | | | |
| --- | --- | --- | --- | --- | --- | --- | --- | --- | --- | --- | --- | --- | --- | --- | --- | --- | --- | --- | --- | --- |
|  | d1 | d2 | d3 | d4 | d1 | d2 | d3 | d4 | d1 | d2 | d3 | d4 | d1 | d2 | d3 | d4 | d1 | d2 | d3 | d4 |
| A |  |  |  |  |  |  |  |  |  |  |  |  |  |  |  |  |  |  |  |  |
| B |  | | | |  |  |  |  |  |  |  |  |  |  |  |  |  |  |  |  |
| C |  |  |  |  |  | | | |  |  |  |  |  |  |  |  |  |  |  |  |
| D |  |  |  |  |  |  |  |  |  | | | |  |  |  |  |  |  |  |  |
| E |  |  |  |  |  |  |  |  |  |  |  |  |  | | | |  |  |  |  |

**Figure S4: HT-29 spheroid eccentricity is largely similar between different plate types, but spheroid diameter may vary.** Tables display statistical significance of Sidak multiple comparisons between values of a) spheroid diameter and b) spheroid eccentricity for plate types and days in culture (d1-d4) as indicated, experimental data as depicted in Figure 5. Colors indicate levels of significance: gray, n.s.; green, p ≤ 0.05; yellow, p ≤ 0.01; orange, p ≤ 0.001; red, p ≤ 0.0001.

## Supplementary Figure 5

**a)**

|  | B | C | D | E | F |
| --- | --- | --- | --- | --- | --- |
| A |  |  |  |  |  |
| B |  |  |  |  |  |
| C |  |  |  |  |  |
| D |  |  |  |  |  |
| E |  |  |  |  |  |

**b)**

|  | B | C | D | E | F |
| --- | --- | --- | --- | --- | --- |
| A |  |  |  |  |  |
| B |  |  |  |  |  |
| C |  |  |  |  |  |
| D |  |  |  |  |  |
| E |  |  |  |  |  |

**c)**

|  | B | C | D | E | F |
| --- | --- | --- | --- | --- | --- |
| A |  |  |  |  |  |
| B |  |  |  |  |  |
| C |  |  |  |  |  |
| D |  |  |  |  |  |
| E |  |  |  |  |  |

**d)**

|  | B | C | D | E | F |
| --- | --- | --- | --- | --- | --- |
| A |  |  |  |  |  |
| B |  |  |  |  |  |
| C |  |  |  |  |  |
| D |  |  |  |  |  |
| E |  |  |  |  |  |

**e)**

|  | B | C | D | E | F |
| --- | --- | --- | --- | --- | --- |
| A |  |  |  |  |  |
| B |  |  |  |  |  |
| C |  |  |  |  |  |
| D |  |  |  |  |  |
| E |  |  |  |  |  |

**Figure S5: HaCaT spheroids primarily vary in frequency of Ki-67+ cells, in volume, and in nuclear volume between different plate types.** Tables display statistical significance of Sidak multiple comparisons between values of a) spheroid volume, b) nuclear count per spheroid, c) fraction of Ki-67+ nuclei, d) density of nuclei packing, and e) volume of individual nuclei for plate types as indicated, experimental data as depicted in Figure 6. Colors indicate levels of significance: gray, n.s.; green, p ≤ 0.05; yellow, p ≤ 0.01; orange, p ≤ 0.001; red, p ≤ 0.0001.

## Supplementary Figure 6

**a)**

|  | B | C | D | E | F |
| --- | --- | --- | --- | --- | --- |
| A |  |  |  |  |  |
| B |  |  |  |  |  |
| C |  |  |  |  |  |
| D |  |  |  |  |  |
| E |  |  |  |  |  |

**b)**

|  | B | C | D | E | F |
| --- | --- | --- | --- | --- | --- |
| A |  |  |  |  |  |
| B |  |  |  |  |  |
| C |  |  |  |  |  |
| D |  |  |  |  |  |
| E |  |  |  |  |  |

**c)**

|  | B | C | D | E | F |
| --- | --- | --- | --- | --- | --- |
| A |  |  |  |  |  |
| B |  |  |  |  |  |
| C |  |  |  |  |  |
| D |  |  |  |  |  |
| E |  |  |  |  |  |

**d)**

|  | B | C | D | E | F |
| --- | --- | --- | --- | --- | --- |
| A |  |  |  |  |  |
| B |  |  |  |  |  |
| C |  |  |  |  |  |
| D |  |  |  |  |  |
| E |  |  |  |  |  |

**e)**

|  | B | C | D | E | F |
| --- | --- | --- | --- | --- | --- |
| A |  |  |  |  |  |
| B |  |  |  |  |  |
| C |  |  |  |  |  |
| D |  |  |  |  |  |
| E |  |  |  |  |  |

**Figure S6: HT-29 spheroids primarily vary in nuclear counts, spheroid volume, and nuclear volume between different plate types.** Tables display statistical significance of Sidak multiple comparisons between values of a) spheroid volume, b) nuclear count per spheroid, c) fraction of Ki-67+ nuclei, d) density of nuclei packing, and e) volume of individual nuclei for plate types as indicated, experimental data as depicted in Figure 7. Colors indicate levels of significance: gray, n.s.; green, p ≤ 0.05; yellow, p ≤ 0.01; orange, p ≤ 0.001; red, p ≤ 0.0001.


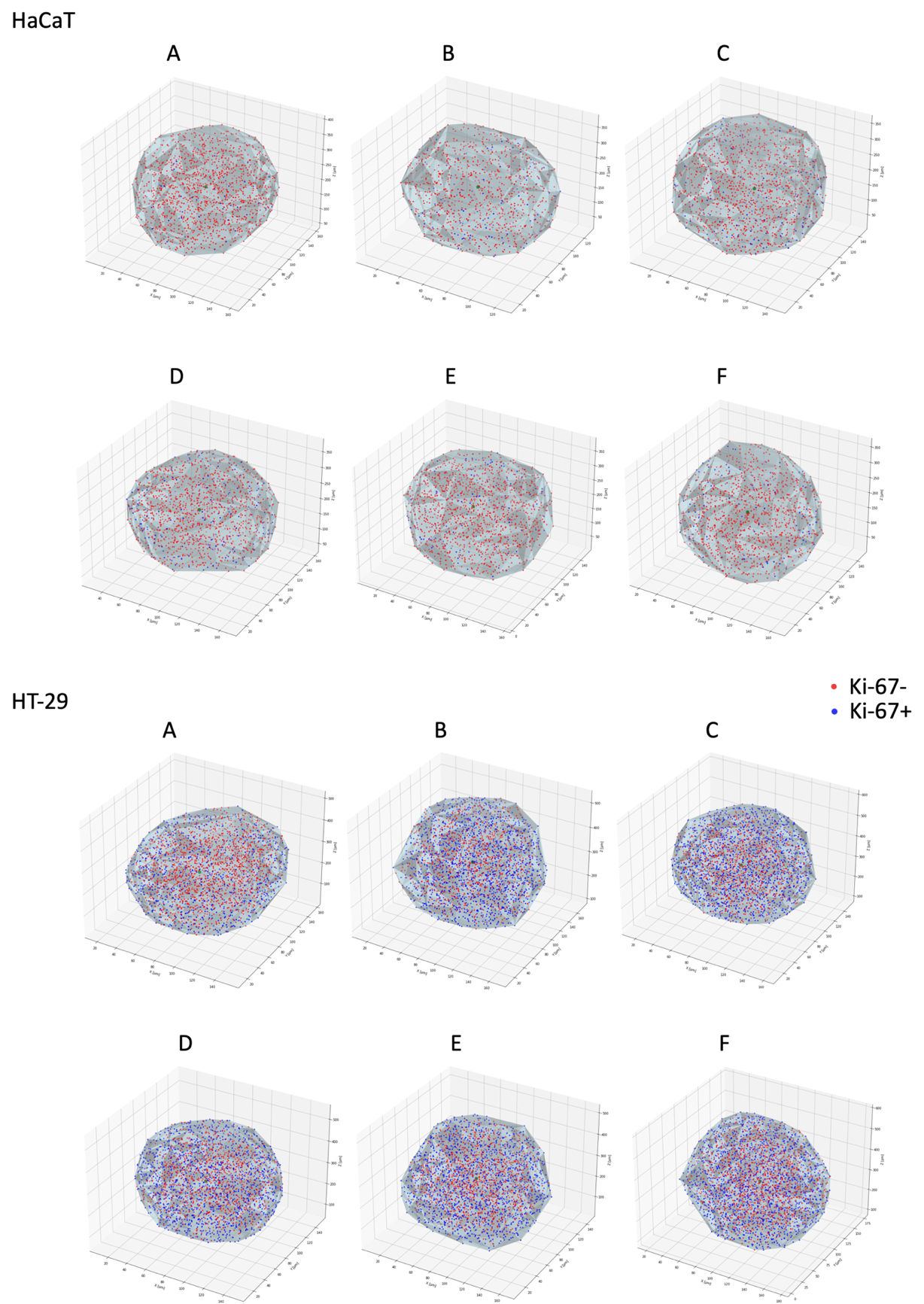


**Figure S7: Frequency of Ki-67+ cells varies between HaCaT and HT-29 cells as well as between different plate types.** 3D-plots show images of the distribution of Ki-67- (red dots) and Ki-67+ cells (blue dots) within individual representative spheroids. Grey hull shows the outline of spheroids, the center of mass of the spheroids is indicated by a green dot. Plate types A-F as indicated.
